# SBA-15 Mesoporous Silica as Catalytic Support for Hydrodesulfurization Catalysts—Review

**DOI:** 10.3390/ma6094139

**Published:** 2013-09-17

**Authors:** Rafael Huirache-Acuña, Rufino Nava, Carmen L. Peza-Ledesma, Javier Lara-Romero, Gabriel Alonso-Núñez, Barbara Pawelec, Eric M. Rivera-Muñoz

**Affiliations:** 1 Facultad de Ingeniería Química, Universidad Michoacana de San Nicolás de Hidalgo Ciudad Universitaria, Morelia, Michoacán 58060, Mexico; E-Mail: lararom@umich.mx; 2 Facultad de Ingeniería, Universidad Autónoma de Querétaro, Centro Universitario, Cerro de las Campanas, Querétaro, C.P. 76010, Mexico; E-Mail: rufino@uaq.mx; 3 Centro de Física Aplicada y Tecnología Avanzada, Universidad Nacional Autónoma de México, A.P. 1-1010, Querétaro, C.P. 76000, Mexico; E-Mail: cpeza@fata.unam.mx; 4 Centro de Nanociencias y Nanotecnología, Universidad Nacional Autónoma de México, Ensenada, Baja California, C.P. 22860, Mexico; E-Mail: galonso@cnyn.unam.mx; 5 Institute of Catalysis and Petrochemistry, CSIC, c/Marie Curie 2, Cantoblanco, Madrid 28049, Spain; E-Mail: bgarcia@icp.csic.es

**Keywords:** SBA-15, mesoporous, silica, catalyst support, hydrodesulfurization

## Abstract

SBA-15 is an interesting mesoporous silica material having highly ordered nanopores and a large surface area, which is widely employed as catalyst supports, absorbents, drug delivery materials, *etc.* Since it has a lack of functionality, heteroatoms and organic functional groups have been incorporated by direct or post-synthesis methods in order to modify their functionality. The aim of this article is to review the state-of-the-art related to the use of SBA-15-based mesoporous systems as supports for hydrodesulfurization (HDS) catalysts.

## 1. Introduction

Nowadays, in the petroleum refining industry, the deep desulfurization of “more dirty” feeds containing refractory S-containing compounds, such as 4,6-dimethyl dibenzothiophene (4,6-DMDBT), is a priority task, due to an increasing demand for ultralow S-containing fuels imposed by more strict environmental regulations [1]. Since it is impossible to achieve deep hydrodesulfurization (HDS) of fuels using classical Co(Ni)Mo(W)/Al_2_O_3_ sulfide catalysts [2], it is urgent to develop new catalysts with higher activities, greater selectivity and better resistance to H_2_S and N-poisoning than those that are being used currently.

The origin of the almost exclusive use of alumina as support has been ascribed to its outstanding textural and mechanical properties and its relatively low cost [3]. However, the presence of undesirable strong metal-support interactions in the alumina-supported catalysts has triggered research devoted to the development of new supports for HDS applications [4,5,6,7,8,9,10,11,12,13]. In this sense, the use of ordered mesoporous siliceous molecular sieves as supports has been intensively investigated [5,13,14].

Ordered mesoporous silicas were first reported in 1992 [15]. Since then, significant progress has been made in their morphology control, pore size adjustment, composition variation and application developments [16,17,18]. During the last two decades, various mesoporous structures have been synthesized, which can be roughly classified into three categories based on the pore types: nearly spherical cage, cylindrical channel and bi-continuous channel [19]. Among different ordered mesoporous silicas, SBA-type silicas are the most frequently studied [13,14,20,21,22,23,24,25,26,27,28,29,30,31,32,33,34,35,36,37,38,39,40,41,42,43,44,45,46,47,48,49,50,51,52,53,54,55,56,57,58,59,60,61,62,63,64,65,66,67,68,69,70,71,72,73,74,75,76,77,78,79,80,81,82]. SBA-15 silica (SBA = Santa Barbara Amorphous) exhibits interesting textural properties, such as large specific surface areas (above 1000 m^2^·g^−1^), uniform-sized pores (in range 4–30 nm), thick framework walls, small crystallite size of primary particles and complementary textural porosity. The advantage of the use of SBA-15 material as support includes also its high surface-to-volume ratio, variable framework compositions and high thermal stability [20,21,22].

**Figure 1:**
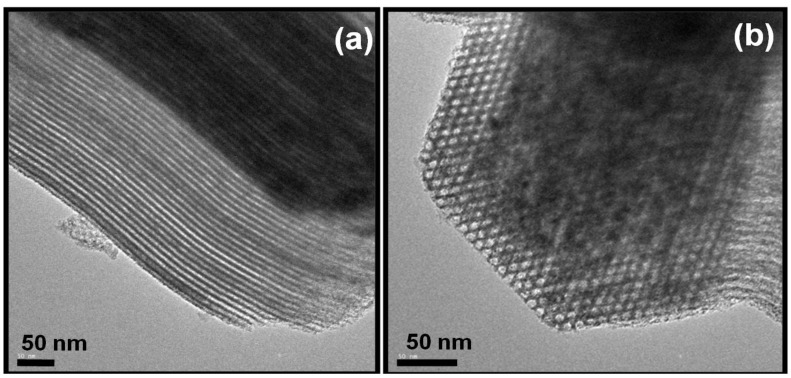
High resolution transmission electron microscopy (HRTEM) micrographs of SBA-15 mesoporous silica. The size and morphology of the highly ordered hexagonal pores in a 2D array (**a**) with long 1D channels (**b**) (*p6mm* plane group) can be observed.

Figure 1a,b shows two high resolution transmission electron microscopy (HRTEM) micrographs of our laboratory-synthetized SBA-15 mesoporous silica calcined at 550 °C. As seen in this figure, SBA-15 shows hexagonal pores in a 2D array with long 1D channels (*p*6*mm* plane group) [21]. The channels are interconnected by small micropores. Thus, SBA-15 exhibits mainly mesoporous structure and possesses a small amount of micropores. The large pore size of this mesoporous material can mitigate the diffusion barrier for the reactants and the products. However, pure siliceous SBA-15 has an electronically neutral framework and lacks Brønsted acidity. This problem could be circumvented by SBA-15 modification in order to make this mesoporous substrate more versatile in terms of its possible applications, either as a structural material or support, in absorption processes, separation, catalysis, *etc.*; or, as reviewed in this case, as support of catalysts used in hydrodesulfurization (HDS) reactions in petroleum refining processes.

There are many approaches to prepare better SBA-15-supported catalysts, such as changing the support properties by substitution of Si^4+^ by different cations, functionalization with different groups, *etc.*, changing the active phase component, varying the preparation method, *etc.* In general, the studies in this field aim to get relationships between different physical and chemical properties of the support and active phases and catalyst performance for hydrotreating reactions, such as hydrodesulfurization (HDS), hydrodenitrogenation (HDN), hydrodeoxygenation (HDO) and/or hydrodearomatization (HDA). Recent revision by Rahmat *et al.* [22] on the SBA-15-based catalysts focused on their application in biorefinery production. In this review, we are going to show the possibilities of the use of SBA-15 silica as support for hydrodesulfurization catalysts.

## 2. Influence of Support 

### 2.1. Synthesis of Bare SBA-15 

The synthesis of SBA-15 molecular sieve requires the use of triblock copolymer (typically non-ionic triblock copolymer) as a structure directing agent and tetramethyl orthosilicate (TMOS), tetraethyl orthosilicate (TEOS) or tetrapropyl orthosilicate (TPOS) as a silica source [22,23,24,25,26]. In a typical synthesis, the structure directing agent (e.g., Pluronic P123: EO_20_PO_70_EO_20 _from BASF) is dissolved under stirring in a solution of water and 2 M HCl. After this, the required amount of tetraethyl orthosilicate (TEOS) is added at 35 °C. Then, this aqueous solution of triblock copolymer and TEOS is kept under stirring conditions for 20 h for aging. At this stage of preparation, the control of pH is of paramount importance, because the formation of ordered hexagonal SBA-15 with uniform pores up to 30 nm might only occur in strong acidic media, *i.e.*, pH ≈ 1 [23,24,25]. In the case when the pH of a solution will be higher than that of the isoelectric of silica, *i.e.*, at pH 2–6, no precipitation or formation of silica gel occurs. The formation of disordered or amorphous silica was observed for the synthesis carried out at neutral pH (≈7) [25].

Zhao *et al.* [23,24,25] reported the synthesis of a variety of mesoporous SBA-type silicas using non-ionic triblock copolymers as template. This type of surfactant is very interesting, because it is easily separated, is nontoxic, biodegradable and inexpensive [23]. The synthesis conducted with these surfactants usually occurs in low-pH solutions (pH ≈ 2), where the interaction occurs through an S^0^H^+^X^−^I^+^ mechanism (S^0^H^+ ^being the surfactant hydrogen bonded to a hydronium ion, X^−^ the chloride ion and I^+^ the protonated silica) [24]. The mixture is subsequently aged at 80 °C overnight. Then, the solid obtained is filtered, washed thoroughly with deionized water, dried first in air at room temperature and then calcination is carried out by slowly increasing temperature from room temperature to 500 °C in 8 h and heating at 500 °C for 6 h. The latter step of the template removal is one of the crucial aspects in the synthesis of ordered mesoporous, because the procedure employed during calcination influenced the final textural properties of SBA-15 material. According to Zhao *et al.* [23], the calcination at 500 °C led to formation of SBA-15 with interlattice *d* spacing of 74.5–320 Å between the (100) planes, pore volume up to 0.85 m^3^·g^−1^ and silica wall thickness of 31–64 Å.

The effect of the synthesis conditions on the textural and structural properties of SBA-15 materials was studied by Klimova and co-workers [26] by using a statistical model built from a full 2^3^ factorial design at two levels. Textural and structural differences induced by change in the synthesis conditions (temperature of the reaction of gel formation, as well as temperature and time of the aging stage) were discussed in terms of the mechanism of SBA-15 formation in the presence of Pluronic P123. The statistical analysis showed that both synthesis and aging temperatures had a significant influence on the textural and structural properties of SBA-15 materials. Their increase affected in a positive way the Brunauer-Emmett-Teller (BET) surface area, total pore volume, pore diameter and unit-cell parameter, producing, simultaneously, a decrease of micropore area and pore wall thickness. As compared to aging temperature, it was found that the gel aging time is of much lower importance, with the exception of micropore area, which continued decreasing with the increase of aging time [26].

### 2.2. Modulation of Pore Diameter

The control of the support’s pore diameter is of paramount importance for SBA-15-based hydrotreating catalysts, which has diffusion limitations of large feed molecules to enter into unidirectional channels of the SBA-15. To study the effect of different pore diameters, the SBA-15-supported catalysts having pore diameters in the range 5–20 nm were screened for hydrotreating of heavy gas oil [27,28,29].

In this direction, Boahene *et al.* [27] tested FeW/SBA-15 sulfide catalysts with pore diameters in the range 5–20 nm as potential hydrotreating catalysts for hydrotreatment of heavy gas oil. The highly ordered siliceous SBA-15 substrates with different pore diameters were synthesized using hexane as a micelle expander under acidic conditions. It was found that the catalyst with a pore diameter of 10 nm was the best among the FeW/SBA-15 catalysts studied, probably due to the sufficient mass transfer of the reactants through the catalyst’s pores, while maintaining a high surface area necessary for metal dispersion [27].

The effect of support pore diameter was reported also for NiMo/SBA-15 [28] and NiMo/Al-SBA-15 [29] sulfide catalysts tested in the hydrotreating of gas oil. A series of binary NiW catalysts supported on SBA-15 with different pore sizes were prepared by Lei *et al.* [28]. The NiW/SBA-15 sulfide catalysts with different pore sizes were tested in the hydrogenation of a heavy oil (distillation temperature: 320–340 °C) derived from the direct coal liquefaction process. It was found that the pore size of the support has a significant influence on the Ni/W crystallite size and catalytic activity, larger Ni–W crystals being formed on the supports having larger pores. As expected, the catalysts with the largest pores displayed the highest HDN and HDA activities for heavy oil upgrading [28].

For the NiMo/Al-SBA-15 catalysts, Chandra Mouli *et al.* [29] employed direct and post synthesis modification methods to incorporate aluminum in the framework of SBA-15. In the direct and post-synthesis approaches, the aluminum sulfate and ammonium hexafluoroaluminate were used as a source of aluminum, respectively. In the direct synthesis, the highest pore diameter was limited to 7 nm. The post-synthesis support modification with the ammonium hexafluoroaluminate led to Al-SBA-15 substrate with pore diameter greater than 10 nm. The pore structure of the synthesized SBA-15 did not collapse until 13 nm of pore diameter, as confirmed from the small angle XRD and TEM analysis. The Ni–Mo/Al-SBA-15 sulfide catalysts with different pore diameters were tested in hydrotreating of heavy gas oil carried out in a trickle bed continuous reactor [29]. It was found that HDS and HDN activities increased with the increase in pore diameter until 13 nm, and then decreased, due to the collapse in the pore structure and poor dispersion of metals on the supports, as evidenced from the BET and TEM analysis. As a consequence, the NiMo/Al-SBA-15 sample prepared by the post-synthesis method exhibited the largest HDS activity in the hydrotreating of heavy gas oil [29].

### 2.3. Redox and Acid-Based Properties 

The pure siliceous SBA-15 material has no Brønsted acidity. Thus, considerable efforts have been already applied to create acid sites in the silica-based materials by the isomorphous substitution of the Si^4+^ atoms by Al^3+^ ions [29,30,31,32,33,34]. Moreover, the substitution of Si^4+^ ions of SBA-15 by foreign ion (Al^3+^, Ti^4+^ or Zr^4+^) is an efficient method to enhance the catalyst stability and leads to catalysts with redox properties [14].

#### 2.3.1. Al^3+^ Ion Loading

SBA-15 support modification with Al^3+^ ions could be achieved by direct [29,30,31,32,33,34] and post-synthesis modification [35,36,37,38,39] methods. Incorporation of Al during the one-pot synthesis presents difficulties, because the high acidity (pH ≈ 1.5) needed for the creation of ordered pore structure of SBA-15 leads to leaching of aluminum and its coordination in the octahedral state. This problem can be circumvented when Al is introduced by the post-synthesis support’s grafting with aluminum isopropoxide in non-aqueous solutions, anhydrous AlCl_3_, ammonium hexafluoroaluminate or sodium aluminate in aqueous solution, followed by calcination [35,36,37,38,39]. In the post-synthesis method, the strong basic solution facilitates the hydrolysis of AlF_6_^3−^ to Al(OH)_4_^−^, and thus, the insertion of Al into the Si–SBA-15 framework. Thus, the adjustment of pH value in the range near 9.0–9.5 is a key procedure for quantitatively incorporating Al into Si–SBA-15.

##### Direct Synthesis

Macías Esquivel *et al.* [30] reported the synthesis of NiMo catalysts supported on Al-SBA-15 using acid Mo precursor solutions to avoid molybdenum reaction with mesopores walls and to preserve the support hexagonal pore arrangement [30]. In addition, when NiW HDS catalysts supported on Al-SBA-15 were prepared using HCl acidified solutions of ammonium metatungstate and nickel nitrate, at pH ≈ 1.0, the integrity of the SBA-15 support was preserved. After calcination, the oxide precursors showed pore diameters in the range 5.4–6.6 nm. The increase in the wall thickness observed after Ni and W impregnation was interpreted as due to covering of the walls of the pores by metals. It was found that aluminum incorporation to the framework of SBA-15 increased the transformation of 4,6-DMDBT, and the product distribution shows that the increase in activity is due to a greater contribution of the isomerization reaction pathway when aluminum is present on the catalyst. The improvement in the overall HDS activity was related to the higher number of coordinatively unsaturated nickel sites associated to molybdenum sulfide promoted by nickel (Ni–Mo–S phase) [30].

The Al-SBA-15 molecular sieve was used also for supporting NiMo sulfide catalysts by Sundaramurthy *et al.* [31]. The support was prepared by conventional hydrothermal method. The catalyst activity evaluated in hydrotreating of light gas oil (LGO) demonstrated that HDS and HDN activities of this catalyst are comparable with the conventional NiMo/Al_2_O_3 _sulfide catalyst under reaction conditions, which approach industrial practice [31]. The impact of Al ions on the dispersion of the support surface on the final catalytic behavior of NiMo(W)/SBA-15 sulfide catalysts was studied also by Olivas and Zepeda [32]. The direct-synthesis method and aluminum isopropoxide as Al precursor were employed for the Al incorporation into the silica framework. The ^29^Si NMR spectra of the calcined NiMo(W)/Al-SBA-15 catalysts demonstrated that this one-pot synthesis method leads to partial incorporation of Al into the silica framework. Al-containing catalysts showed significant changes in the catalytic properties of the NiMo(W) sulfide catalysts in both HYD and HDS reactions with respect to the Al-free counterpart. In general, Al-incorporation led to an enhancement of the hydrogenation ability of the catalysts, and this hydrogenation ability was related to the acidity of the catalysts [32].

Muthu Kumaran *et al.* [33] studied the effect of Si/Al ratio of Al-SBA-15 substrate on the catalytic functionalities of hydrotreating Mo and Co(Ni)Mo catalysts. Al-SBA-15 substrates with varying Si/Al ratio (Si/Al ratio of 10, 20, 30 and 40) were prepared by direct synthesis method using Al-isopropoxide as the Al source. Concerning the effect of Si/Al ratio, the authors concluded that the molybdenum dispersion and anion vacancies, as well as catalytic activities are significantly influenced by Al content. The high Mo dispersion on isolated Al sites in Al-SBA-15 and the consequent increase of anion vacancies at the edge sites of Mo as a function of Si/Al ratio appears to be responsible for the outstanding activities of Al-SBA-15-supported catalysts [33].

In another study by the same authors [34], the Al-SBA-15 (Si/Al ratio = 10) substrate was used for supporting Mo, CoMo and NiMo catalysts. The effect of Mo loading and promoter incorporation (Co *vs.* Ni) was investigated. The catalyst characterization by ^27^Al NMR and IR spectroscopy indicated that Al was incorporated mainly into the SBA-15 structure [34]. The mean pore diameter for bare SBA-15 was 6.6 nm, while for Al-SBA-15-based materials, it was close to 7.8 nm. A comparative study of the reducibility of 8% Mo/SBA-15 and 8% Mo/Al-SBA-15 (Si/Al ratio = 10) catalysts by the temperature-programmed reduction (TPR) technique suggested a stronger interaction of Mo on the latter compared to the former sample [33]. As a consequence of the combined effects of the stronger metal-support interaction on the Al-SBA-15 substrate and the effect of promoter ions, the SBA-15-supported catalysts exhibited higher reducibilities than its Al-SBA-15-supported counterparts [34]. These differences in reducibility had a bearing effect on catalytic HDS activity, binary samples supported on SBA-15 being more active in the HDS of the thiophene reaction. On the contrary, all sulfided catalysts supported on Al-SBA-15 substrate showed outstanding activity for hydrogenation [33,34]. From the oxygen chemisorption-activities correlation, it was concluded that the oxygen chemisorption was not specific to any one of the catalyst’s functionalities (HDS *vs.* HYD) [34].

##### Post-Synthesis Grafting

Both direct and post-synthesis methods were used by Chandra Mouli *et al.* [29] for the preparation of Al-SBA-15-suported NiMo catalysts (nominal SiO_2_/Al_2_O_3_ molar ratio of 20). In the direct synthesis method, when the amount of HCl in the synthesis gel is reduced, the aluminum content in the final product was larger. This was attributed to pH of the synthesis gel under, which alumina is condensed to form primary structural building units, which further leads to the secondary building units. The post-synthesis support grafting with Al was carried out in the pH range of 9.0 ± 0.5 using ammonium hexafluoroaluminate as Al precursor. During this process, the Al was incorporated in the framework of SBA-15, which has helped in achieving required acidity and pore diameter. The post-synthesis method led to catalysts with much higher pore diameter (in the range 10.6–18.2 nm) than those achieved using the direct synthesis method (7.0 nm). As a consequence, the NiMo/Al-SBA-15 sulfide catalysts prepared by the post-synthesis method exhibited higher activity in the hydrotreating of heavy gas oil than their counterparts prepared by the direct synthesis method [29].

The post-synthesis method of SBA-15 grafting with Al was employed by Macías Esquivel *et al.* for the preparation of NiMo/Al-SBA-15 catalysts [30,35]. To obtain Al-SBA-15 supports with Si:Al atomic ratio of 15 and 30, the required amount of SBA-15 was dispersed in an anhydrous hexane containing different amounts of aluminum isopropoxide. Studies demonstrated that highly ordered mesoporous Al-SBA-15 with high aluminum content and high hydrothermal stability could be synthesized by a pH-adjusting and high-temperature hydrothermal treatment approach. The catalyst characterization results showed that both Al-SBA-15 substrates exhibited homogeneously distributed Al species in the walls, and the pore diameter was about 6.6 nm [30,35]. The catalytic response of NiW/Al-SBA-15 sulfide catalysts was evaluated in HDS of 4,6-DMDBT reaction. The activity results demonstrated that, as compared with γ-Al_2_O_3_- and SBA-15-supported catalysts, the NiW/Al-SBA-15 sulfide catalysts showed outstanding HDS activity [35]. This enhancement of HDS activity was explained by the authors as being due to the presence of Brønsted acid sites, a higher dispersion of the WS_2_ phase and the presence of a larger amount of coordinatively unsaturated sites (CUS) in the sulfide catalysts [30,35].

The effect of Si/Al ratio of Al-SBA-15 supports prepared by the post-synthesis method was investigated by Klimova *et al.* [36]. It was found that that HDS activity of NiMo sulfide catalysts increases with Al incorporation in the SBA-15 support, reaching a maximum at a Si/Al molar ratio of 20. This was explained in terms of the high dispersion of Ni and Mo active phases and the bifunctional character of these catalysts, namely, participation of Brønsted acid sites on the support in the catalytic transformations of 4,6-DMDBT prior to its desulfurization [36].

The study by Gómez-Cazalilla *et al.* [37] on the catalytic response of chromium sulfide supported on Al-SBA-15 demonstrated that post-synthesis support alumination highly modifies the nature of the acidic properties of the support, providing a stronger metal-support interaction. As a consequence, better dispersion of the chromium species is obtained. Thus, the Al-SBA-15-supported chromium sulfide catalysts exhibit good activity in the HDS of DBT reaction under high hydrogen pressure [37].

The catalytic performance of noble metals supported on SBA-15 and Al-SBA-15 in the HDS of thiophene was also reported [38,39]. Al-SBA-15 substrates were prepared by a grafting method using aluminum isopropoxide (Al(OC_3_H_7_)_3_) hexane solution. The Pt/Al-SBA-15 exhibited higher HDS activity than SBA-15-based ones, and its activity was higher than that of commercial CoMo/Al_2_O_3_ HDS catalyst [38,39]. Both the catalyst acidity and Pt dispersion on Al-SBA-15 were remarkably higher than on SBA-15. On the basis of an FT-IR study of thiophene adsorption on Al-SBA-15, it was concluded that the interaction between the thiophene molecule and the Brønsted acid sites of the Al-SBA-15 substrate was stronger than those of SBA-15. A possible reaction mechanism proposed by Kanda *et al.* [39] for the highly active Pt/Al-SBA-15 involves hydrogen activation on Pt particles to form spillover hydrogen and thiophene activation on the Brønsted acid sites of Al-SBA-15 substrate.

#### 2.3.2. Ti^4+ ^Ion Loading

Similarly to the support modification with Al, the HDS activity of the SBA-15-based catalysts could be enhanced by the incorporation of Ti^4+^ ion into SBA-15 [13]. This is usually archived via a direct synthesis method or by post-synthesis grafting procedure. The latter method is generally used to create dispersed and isolated Ti-species for the catalyst use in either photocatalysts or oxidation catalysis. However, for the HDS catalysis, the best method of the Ti incorporation is via direct hydrothermal synthesis procedure, which involves the addition of a titanium source, such as titanium isopropoxide (Ti(OPr*^i^*)_4_) in ethanol or titanium ethoxide (Ti(OEt)_4_) in H_2_O_2_, to the gel [13].

##### Direct Synthesis

The direct synthesis method was employed by Olivas and Zepeda [32] for Ti^4+^ ions incorporation into the framework of the SBA-15 substrate. Ti-SBA-15-supported NiMo and NiW sulfide catalysts exhibited the enhancement of the activity in HDS and HDN reactions with respect to Ti-free counterparts [32]. This is because an enhancement of the active phase dispersion leads to an increase of the support acidity. Interestingly, both NiMo and NiW catalysts modified with Ti were found to be more active than their Al-containing counterparts. This is probably because, contrary to Al^3+^, the Ti incorporation into the framework of SBA-15 was complete, as confirmed by ^29^Si NMR. As expected, the W-based catalysts showed higher activity in both hydrotreating reactions than their Mo-based counterparts [32].

Similarly to the work by Olivas and Zepeda [32], the incorporation of Ti into the SBA-15 by the direct synthesis method was reported to be beneficial for morphology and HDS activity of CoMo sulfide catalysts, providing better dispersion for the oxide and sulfide metal species, which, in turn, favored the sulfidation of cobalt species [40]. The bifunctional CoMo/Ti-SBA-15 catalysts exhibited higher activities in the HDS of dibenzothiophene reaction than their Ti-free counterparts. The reaction proceeds mainly via the direct desulfurization (DDS) reaction route and the hydrogenation reaction pathway. The Ti incorporation into SBA-15 led to the enhancement of the direct desulfurization route of this reaction. Interestingly, from the catalyst characterization results, it was concluded that the presence of small clusters of the TiO_2 _anatase phase located on the SBA-15 support surface together with the Ti^4+^ ions incorporated into the framework structure of the SBA-15 are both beneficial factors influencing the desulfurization properties of the CoMo/Ti-SBA-15 catalysts [40], in good agreement with a study by Lizama *et al.* [41] on the NiMo catalysts supported on hybrid TiO_2_-SBA-15 materials.

##### Post-Synthesis Grafting

The catalyst characterization results presented by Laniecki and Wojtowski [42] suggest that Ti-SBA-15 could be appropriate support for molybdenum-based hydrotreating catalysts. The Ti-SBA-15 supports were prepared by post-synthesis grafting of the SBA-15 with titanium isopropoxide as Ti source. The textural properties of SBA-15 were preserved, and only Lewis acid sites were created upon incorporation of Ti by the post-synthesis method. The use of different sources of Mo (ammonium heptamolybdate and molybdenum carbonyl) leads to formation of different molybdenum oxide species. On the basis of the results of the characterization of oxide catalyst precursors, the authors deduced that catalyst activation by sulfidation or nitridation might lead to bifunctional catalysts active in HDS, HDN or hydrocracking reactions [42]. This was confirmed later by Gutiérrez *et al.* [43] for NiMo/Ti-SBA-15 catalysts tested in HDS of 4,6-DMDBT reaction. The Ti-containing SBA-15 solids were prepared with varying Ti loading (up to 19 wt % of TiO_2_) by a post-synthesis grafting procedure. In good agreement with the study by Laniecki and Wojtowski [42], the catalyst characterization by different techniques confirmed that this method of Ti incorporation onto SBA-15 led to highly dispersed titanium species without substantial loss of SBA-15 textural characteristics. The catalysts supported on SBA-15 grafted with Ti exhibited a stronger metal-support interaction and a better dispersion of the oxidic and sulfided metal species than its Ti-free counterpart.

#### 2.3.3. Zr^4+^ Ion Loading

Not only Al^3+^ and Ti^4+^, but, also, Zr^4+ ^can be effectively used for dispersing ZrO_2_ by direct, as well as post-synthesis methods [44,45,46,47], and the resultant materials are good supports for the HDS catalysts.

##### Direct Synthesis

The resistance of NiMo and NiW catalysts supported on Zr-SBA-15 and γ-alumina on the poisoning of HDS active sites by quinoline was studied by Soriano *et al.* [44]. The catalysts were tested in simultaneous hydrodesulfurization of DBT and 4,6-DMDBT in the absence and presence of quinoline or naphthalene. NiMo and NiW catalysts supported on Zr-SBA-15 showed higher HDS activity than those supported on γ-Al_2_O_3_. As expected, in the presence of quinoline, all catalysts lost their activity for HDS of 4,6-DMDBT, due to competitive adsorption of quinoline on the active sites. Naphthalene addition had a small effect on NiMo/Zr-SBA-15 catalyst and almost none on NiW/Zr-SBA-15 [44].

Grag *et al.* [45] observed that low temperature reducible molybdenum species are preferentially formed on low ZrO_2_ content SBA-15 supports (10 and 25 wt %). These low temperature reducible Mo and CoMo species exhibit a correlation with catalytic activity on ZrO_2_-containing SBA-15 supports, suggesting that the added ZrO_2 _enhances the formation of low temperature reducible species, which appears to be responsible for catalytic activity. In addition, it was observed that the presence of ZrO_2_ species on the SBA-15 surface significantly improved the hydrothermal stability of the material [45].

##### Post-Synthesis Grafting

Klimova and co-workers used SBA-15 substrate grafted with Zr^4+^ for supporting Mo and NiMo sulfide catalysts [46,47]. Zr-containing SBA-15 materials with high Zr loading (22.4 wt %) were prepared by chemical grafting without considerable degradation of the initial SBA-15 pore structure. The catalyst characterization by different techniques demonstrated that isolated tetrahedral Zr species were mainly present on the support surface. The metal-support interaction increased after support modification with Zr. NiMo catalysts supported on Zr-SBA-15 show high activity in HDS of 4,6-DMDBT, and their activity increases almost linearly with an increase of Zr loading in the support, reaching a maximum at 22.4% of ZrO_2_ loading. This was explained as being due to high hydrogenation activity and promotion of the HYD route of 4,6-DMDBT transformation over NiMo/Zr-SBA-15 sulfide catalysts with respect to the Zr-free NiMo/SBA-15 counterpart [46].

In the other work by the same authors [47], a series of NiMo catalysts with different MoO_3_ loadings (6–30 wt %) was prepared using SBA-15 material covered with ZrO_2_-monolayer as a support. It was observed that ZrO_2_ incorporation on the SBA-15 surface improves the dispersion of the Ni-promoted oxidic and sulfided Mo species, which were found to be highly dispersed, up to 18 wt % of MoO_3_ loading. Further increase in metal charge resulted in the formation of MoO_3_ crystalline phase and an increase in the stacking degree of the MoS_2_ particles. All NiMo catalysts supported on ZrO_2_-modified SBA-15 material showed high activity in the HDS of 4,6-dimethyldibenzothiophene. The best catalyst, having 18 wt % MoO_3_ and 4.5 wt % NiO, was almost twice more active than the reference NiMo/γ-Al_2_O_3_ catalyst [47].

Biswas *et al.* [48] demonstrated that by modification of the SBA-15 substrate with zirconia, an optimum level of surface acidity and metal-support interaction can be archived. The Zr-SBA-15 substrates (Zr/Si ratio = 20 and 40) were prepared by the direct synthesis and the post-synthesis methods, whereas NiMo/Zr-SBA-15 catalysts were prepared by an incipient wetness impregnation technique. Characterization of support confirmed that Zr was incorporated in a framework of the SBA-15 substrate without significant changes in its porous structure. The NiMo/Zr-SBA-15 sulfide catalysts prepared by both the post-synthesis and the direct synthesis methods exhibited larger HDS and HDN activities during hydrotreating of heavy gas oil derived from Athabasca bitumen compared to γ-Al_2_O_3_- and SBA-15-supported counterparts. The largest activity exhibited NiMo/Zr-SBA-15 sulfide catalyst prepared by the post-synthesis method. This was explained by the authors as being due to the combined effects of a larger pore diameter (10 nm), higher Zr loading (23 wt %), higher acidity and a better dispersion of Mo active phases. Moreover, the higher activity of this sample with respect to a commercial NiMo/γ-Al_2_O_3_ catalyst was attributed to the uniform mesoporous structure and optimum metal-support interaction of the NiMo/Zr-SBA-15 catalyst [48].

#### 2.3.4. Comparison of SBA-15-Loaded with Different Cations (Al^3+^, Ti^4+^ and Zr^4+^)

Gutiérrez *et al.* [49] used SBA-15 doped with ZrO_2_ and TiO_2_ for supporting Mo HDS catalysts. SBA-15 materials with different TiO_2_ or ZrO_2_ loadings were prepared by a chemical grafting procedure, and Ti and Zr oxide species were found well dispersed on SBA-15 surface. The textural characterization of the supports indicated that the incorporation of Zr or Ti oxides in the SBA-15 surface led to a decrease in the SBA-15 textural properties. This decrease is larger for the Zr-containing material, because of higher zirconia weight loading. The dispersion of Mo oxide species increases with TiO_2_ or ZrO_2_ loading in the SBA-15 support. Catalytic activity tests in the HDS of 4,6-dimethyldibenzothiophene showed that the modification of SBA-15 supports with Ti and Zr species significantly improves the performance of unpromoted Mo catalysts in this reaction [49].

Romero-Pérez *et al.* [50] used Al and Zr as stabilizing agents for RuS_2_-pyrite phase supported on SBA-15. RuS_2_ catalysts supported on SBA-15 and Zr-SBA-15 demonstrated higher activity in the HDS of the DBT reaction than that supported on Al-SBA-15, with conversions close to 100% at a reaction temperature of 360 °C. The catalysts sulfided at 500 °C showed larger activities, because of the formation of a greater quantity of the RuS_2_-pyrite structure and a higher dispersion of the active phase [50].

Chandra Mouli *et al.* [51] prepared NiMo catalysts supported on SBA-15 modified with different heteroatoms (Ti, Zr and Ti + Zr). The catalytic activities were evaluated in the hydrotreating of light gas oil (LGO) carried out in a down flow trickle bed reactor. It was found that the key difference between the various catalysts is the Mo interaction with the substituted heteroatom, and the nature of this heteroatom had a great impact on the molybdenum phases formed after catalyst calcination. Mo L_3_ edge XANES analysis of the catalysts, along with the Raman and XRD results, showed that Zr-SBA-15 support is able to disperse the molybdenum oxide phase much better than the other supports modified with Ti and Ti + Zr. The optimum strength of the Zr–O–Mo interaction resulted in the formation of a well dispersed MoO*_x_* phase, which exhibited good catalytic properties upon sulfidation. The HDS and HDN activities followed the order: NiMo/Zr-SBA-15 > NiMo/Ti-SBA-15 > NiMo/TiZi-SBA-15 > NiMo/SBA-15. The hydrotreating activities were in good agreement with the catalyst characterization results, NiMo/Zr-SBA-15 and NiMo/Ti-SBA-15 catalysts being the most active in both HDS and HDN reactions, respectively [51].

In general, the catalysts supported on SBA-15 modified with Al^3+^, Ti^4+^ and Zr^4+^ showed a higher HDS activity than their counterparts supported on conventional Al_2_O_3_. For example, NiMo/Al-SBA-15 sulfide catalysts tested in hydrotreating of gas-oil demonstrated HDS and HDN activities comparable with a conventional NiMo/Al_2_O_3_ sulfide catalyst [32], whereas NiMo(W)/Zr-SBA-15 catalyst showed higher HDS activity than those supported on Al_2_O_3_ ones [44].

#### 2.3.5. Surface Functionalization with Different Groups

The increase of the acidity of SBA-15 substrate could be achieved also by support grafting with phosphate. Indeed, in the past, this approach was the most frequently employed method to improve the HDS activity of the Co(Ni)–Mo(W)/Al_2_O_3_ sulfide catalysts [52]. In this direction, the effect of support modification with variable amounts of phosphorus on the HDS activity of Co–Mo–W sulfide catalysts was studied by Huirache-Acuña* et al.* [53]. The sulfided catalysts were tested in the HDS reaction of DBT at 350 °C and 3.1 MPa of total H_2_ pressure. Regardless of the support, it was found that the presence of phosphorous inhibited the HDS activity. Both P-free CoMoW/SBA-15 and CoMoW/SBA-16 catalysts were more active than a CoMo/Al_2_O_3_ commercial catalyst containing a small amount of phosphorous in its formulation [53].

La Parola *et al.* [54] studied the effect of support functionalization with mercaptopropyl groups on the HDS activities of CoMo/SBA-15 and CoMo/HMS sulfide catalysts. The catalysts activity was evaluated in the HDS of thiophene. The effect of the two different silica textures on the catalyst activity was rather small. However, a significant enhancement of the catalytic activity was observed for the catalysts supported on the functionalized silicas. Taking into account the structural and spectroscopic results, the superiority of the thiol-containing catalysts was attributed to the improved dispersion and reducibility of the precursor oxide species [54].

#### 2.3.6. Composite Substrates

In general, the HDS catalysts supported on the hybrid carriers showed advantages in comparison with their pure oxide counterparts (SBA-15 silica, ZrO_2_, TiO_2, _zeolite Beta and γ-alumina), when used as supports to prepare Mo and Ni(Co)Mo sulfide catalysts [41,55,56,57,58,59,60,61]. However, the final catalytic response strongly depends on the composition of the hybrid carrier. This is because the catalyst acidity, pore diameter, specific surface area and metal-support-interaction are the sum of the individual characteristics of each support’s substrate.

Micro-mesoporous composite material β-SBA-15 with the β structure and SBA-15 mesoporous structure was synthesized and used as catalyst support for the HDS of the DBT by Zhang *et al.* [55]. The characterization results demonstrated that NiMo/β-SBA-15 had similar acidity to NiMo/Beta and possessed more acid sites and stronger acidity than NiMo/SBA-15 and NiMo/Al_2_O_3_. Activity evaluation results showed that NiMo/β-SBA-15 exhibited the highest DBT HDS activity among all the studied catalysts and the DBT conversion on NiMo/β-SBA-15 was about 1.6-times as much as that on NiMo/Al_2_O_3_ at a weight time of 0.75 g·min·mol^−1^. The better catalytic performance of β-SBA-15-supported catalyst was attributed to the superiorities of the pore structure and the large amounts of acid sites of the micro-mesoporous β-SBA-15 [55].

The substrates composed of titania deposited on SBA-15 were used by Nguyen Dinh *et al.* [56] for supporting CoMo catalysts. The catalysts were prepared with titanium oxide loadings varying between 10 and 40 wt % up to 30 wt % of TiO_2_; titania was present as highly dispersed anatase nanocrystals in the silica pores, while some segregated titania particles were detected outside the silica network at higher loading (e.g., 40 wt %) [56]. For all the samples, characterization evidenced the preservation of the mesoporous structure of SBA-15 after titania deposition, with open porosity, high surface area, large pore size and pore volume. Mo-based oxidic precursors with molybdenum oxide contents of 20 wt % were then prepared and characterized. Raman spectroscopy evidenced well dispersed polymolybdate species on all the solids, whereas bulk MoO_3_ was also observed on the low TiO_2 _loading supports. This suggested that high TiO_2_ loading is necessary to maintain high molybdenum dispersion. A cobalt-molybdenum catalyst deposited on the composite containing 20 wt % of TiO_2_ was then tested in thiophene HDS; its performance was found to be superior to those of catalysts based on pure TiO_2 _and pure SiO_2_, highlighting the beneficial effect of titanium oxide deposited in the form of nanocrystals inside the mesopores of a SBA-15 support [56].

The effect of core-shell covering of SBA-15 by ZrO_2_ was studied by Klimova and co-workers [57,58,59,60]. ZrO_2_-SBA-15 supports with different ZrO_2_ loadings (12.5, 25, 37.5 and 50 wt %) were prepared by incipient wetness impregnation of pure siliceous SBA-15. ZrO_2_ species were found to be well-dispersed on the SBA-15 surface at ZrO_2_ loading below 25 wt % [57]. At higher ZrO_2_ loadings, the formation of tetragonal and monoclinic crystalline zirconia phases was observed. In the catalysts supported on ZrO_2_-SBA-15 materials, the dispersion of Mo oxide species was found to be much better than on corresponding pure SBA-15 and ZrO_2_. It was found that the dispersion of Mo species increased with the addition of the Ni promoter to both Mo/SBA-15 and Mo/ZrO_2_-SBA-15 samples, while the effect of the cobalt promoter was enhanced when the ZrO_2_-containing SBA-15 support was used, probably due to a stronger Co-zirconia interaction [58]. The sulfided catalysts tested in the HDS of 4,6-DMDBT [57], DBT [58,59] and simultaneous HDS of DBT and 4,6-DMDBT [59] exhibited larger HDS activity than all other samples, including reference Mo/γ-Al_2_O_3 _[58], the NiMo/ZrO_2_-SBA-15 catalyst being the most active among the catalysts studied [57,58,59,60].

### 2.4. Comparison of SBA-15 with Other Supports

In an attempt to develop new more effective mesoporous silica support, the HDS activity of SBA-15-supported catalysts were compared with those supported on hexagonal mesoporous silica (HMS) [54,61] and SBA-16 [53] showing different morphology and pore diameter. The SBA-15 possesses significantly larger pore diameter than those of HMS and SBA-16, and both SBA-15 and SBA-16 substrates are more stable than HMS, due to their thicker pore walls [61].

A comparative study between HMS- and SBA-15-supported CoMo sulfide catalysts was reported by Nava *et al.* [62]. The surface properties of both HMS and SBA-15 silicas correlated well with the HDS activity of sulfided CoMo catalysts, and textural data of the oxide catalysts confirmed that the support’s sequential impregnation with Mo and Co salt solutions, followed by calcination, did not destroy the mesoporous structure of these substrates. Interestingly, it was found that the catalytic response of HMS- and SBA-15-supported catalysts in the HDS reaction of DBT was similar [62]. This was a surprising result, because, in comparison with the HMS, the SBA-15 showed a larger pore diameter. The similar HDS activity of both HMS- and SBA-15-based catalysts might indicate that the wormhole mesostructure of HMS offers similar transport for reactants and products than uniform tubular channels of SBA-15 [62].

In good agreement with a study by Nava *et al.* [62], the small effect of the two different silica textures on the HDS activity of CoMo sulfide catalysts supported on HMS and SBA-15 functionalized with mercaptopropyl groups was observed by Venezia and co-workers [54]. However, the study by Dimitrov *et al.* [63] suggested that SBA-15 should be more effective as support than its HMS counterpart. The activity trend reported by those authors for NiW catalysts, tested in the HDS of thiophene, is: NiW/W-SBA-15 > NiW/W-HMS > NiW/γ-Al_2_O_3_. Since the type of active phases (CoMo *vs.* NiW) and precursors (heptamolybdate against heteropoly-acids) are different in these two works and as it was demonstrated that channels of the SBA-15 could be expanded in order to accommodate metal sulfide phases [64,65], it cannot be said what support is better for supporting HDS catalysts.

The effect of support morphology (SBA-15 against SBA-16) on the HDS activity of ternary Co–Mo–W sulfide catalysts was studied by Huirache-Acuña* et al.* [53]. The morphology of the SBA-15 and SBA-16 is very different. Thus, contrary to the SBA-15 and HMS, SBA-16 has a 3D cubic arrangement of mesopores, corresponding to *Im3m* space group symmetry. This might provide a more favorable mass transfer of reactants and products than in the unidirectional pore system of the SBA-15. The sulfided catalysts were tested in the HDS reaction of DBT, and it was found that catalyst activity did not depend on the support morphology. In terms of the reaction rate constants (*k*) and DBT conversion, both CoMoW/SBA-15 and CoMoW/SBA-16 catalysts were more active than a CoMo/Al_2_O_3_ commercial catalyst containing a small amount of phosphorous in its formulation [53].

## 3. Influence of Active Phase 

A bifunctional catalyst is required for hydrodesulfurization reaction. Since bare SBA-15 has no HDS activity, it is necessary to create active sites. The classical active phases working in the HDS reaction are transition metal sulfides. Transition metal sulfides can be obtained by sulfidation of oxide catalyst precursors or by direct decomposition of different thiosalts. Recently, a few studies on the SBA-15-suported metal phosphides and carbides are reported also, because of their high activity in the HDS reaction.

### 3.1. Oxide Precursors 

#### 3.1.1. Effect of Impregnation pH

The most commonly used method for the preparation of oxide catalyst precursors is support impregnation with the Mo(W) and/or Ni(Co) salt precursors according to the incipient wetness method and employing aqueous solutions of nickel and/or cobalt nitrates, ammonium heptamolybdate and/or ammonium metatungstate. It is known that the catalyst preparation method (co-impregnation or successive impregnation) and type of active phase precursor used have great importance for the final catalyst behavior. Concerning SBA-15-based HDS catalysts, the studies on the influence of catalyst preparation method or type of active phase precursor on the final HDS activity are scarce [66]. The significant changes in the structure and texture of SBA-15 induced by pH of the solutions used for support impregnation were observed by Rayo *et al.* [66]. It was found that impregnation under basic conditions, using NH_4_OH solutions, led to destruction of the hexagonal pore’s ordering and to significant dissolution of the pore walls, causing an important drop in specific surface area and pore volume. Besides, the pH of the impregnating solution influences on the nature of the Ni and Mo surface species. NiMoO_4_ phase was formed in significant amounts for catalysts prepared at basic pH, with NH_4_OH solutions and, to a lesser extent, for the catalyst prepared dissolving the salt precursors in an aqueous solution (pH = 4.5). The changes induced by the pH of the impregnating medium did affect also the final state of the “NiMoS” active phase, leading to important differences in HDS activity of catalysts prepared under basic and acidic conditions. For catalyst prepared in acidic conditions, the structural integrity of SBA-15 was preserved, NiMoO_4_ phase was not formed and the sample exhibited high HDS activity [66].

#### 3.1.2. Effect of Chelating Agent 

It is well demonstrated in the literature that HDS catalysts prepared with chelating agents, such as citric acid or ethylenediaminetetraacetic acid (EDTA), showed higher HDS and HYD activities than those prepared without chelating agents [67,68,69,70,71]. Moreover, the use of chelating agents in the preparation of HDS catalysts led to modification of the type of active sites needed for the hydrogenation and direct desulfurization routes of DBT and 4,6-DMDBT HDS reactions [67,68,69,70,71]. However, the appropriate experimental conditions are needed for coordinate metals by chelating agents, as it was demonstrated recently by Klimova and co-workers [70]. Using those appropriate conditions, the addition of chelating agent into impregnation solution led to an increase of solubility and stability of the metal precursors, leading to further enhance of active metal dispersion and sulfurization degree [70]. Thus, the chelating agents are usually employed with the aim to enhance promotion of Co(Ni)Mo(W) phases [67,68,69,70,71]. Concerning the SBA-15-supported HDS catalysts, the effects of the use of citric acid [67,68,69,70] and EDTA [71] only were reported.

To enhance promotion of NiMoS phase, the use of citric acid as chelating agent was extensively explored by Klimova and co-workers for the preparation of NiMo/SBA-15 sulfide catalysts [67,68,69,70]. In general, it was found that Ni and Mo oxide species were well dispersed in all catalysts prepared with citric acid. Their catalysts prepared with citric acid exhibited higher activity in the HDS of DBT [68,69,70] and in the simultaneous HDS of the DBT and 4,6-DMDBT [70] reactions than their counterparts prepared without using this chelating agent. With the aim of getting a deeper insight with respect to the thermal treatment, the catalysts were prepared by simultaneous impregnation of Ni and Mo species and citric acid using impregnation solutions of acidic or basic pH values (pH = 1 or 9, respectively), followed by drying or calcination at 500 °C in air atmosphere [70]. It was found that the calcination at 500 °C under air atmosphere, before catalyst activation by sulfidation, modified the values of specific rate constants, as well as the proportion of the corresponding HYD and DDS active sites [70]. Both dried and calcined catalysts prepared at pH = 1 were more selective toward the hydrogenation (HYD) route of HDS reaction. For the catalysts prepared from basic solutions (pH = 9), the selectivity in the HDS reaction was strongly affected by the thermal treatment: dried catalyst was highly selective for the direct desulfurization (DDS) of DBT, whereas the calcined one was for the HYD route. Interestingly, the catalysts prepared with citric acid contained a small amount of carbon [68,69].

A beneficial effect of the use of ethylenediaminetetraacetic acid (EDTA) as chelating agent was reported also by Badoga *et al.* [71] for the preparation of NiMo/SBA-15 sulfide catalysts. The NiMo/SBA-15 catalysts prepared with different EDTA/Ni molar ratio were tested in hydrotreatment of Athabasca bitumen-derived light gas oil. As compared with NiMo/SBA-15 and NiMo/γ-Al_2_O_3 _prepared without EDTA, the catalysts prepared with this organic compound showed enhancement of activity. Characterization by XANES pointed out the delayed Ni^2+^ sulfidation (to the temperatures where molybdenum already started to sulfide) as the main cause of improvement in HDS and HDN activity of the catalysts prepared in the presence of organic chelates. Nickel sulfidation starts when the EDTA complex decomposes. As a result, nickel atoms released by the chelating agent can move to the reactive edges of the MoS_2_ to form a finely dispersed sulfide Ni–Mo–S type II active phase. From XRD and Raman analyses, it was observed that with increasing EDTA/Ni molar ratio, the amount of Ni available to aid molybdenum dispersion decreases and the size of MoO_3_ crystallites increases, leading to a poor dispersion [71].

### 3.2. Non-Noble Metal Sulfides 

#### 3.2.1. Catalyst Activated by Sulfidation

Concerning the SBA-15-supported transition metal sulfide catalysts prepared by sulfidation of oxide precursors, with the exception of CrRu/SBA-15 [35] and RuS_2_/Zr-SBA-15 [45], the optimization and testing of Mo/SBA-15 and W/SBA-15 sulfide catalysts in HDS reactions is mainly reported [65,72,73,74,75,76,77,78,79,80,81,82]. The latter catalysts are obtained by sulfidation of their oxide catalyst precursors with a 10% H_2_S/H_2_ gas mixture at 400 °C (Mo/SBA-15) or 450 °C (W/SBA-15) for 4–6 h. The temperature necessary for sulfidation of W-based catalysts is higher than those needed for their Mo-based counterparts, because the WO_3_ convers much slower to WS_2 _than MoO_3_ does to MoS_2_. The preferred phase exhibiting by far the highest activity consists of Ni or Co atoms edge-decorated Mo(W)S_2_ slabs. These phases are commonly referred to as NiMoS, CoMoS, NiWS or CoWS phases. In addition, the less active bulk phases Ni_2_S_3_ or Co_9_S_8_ may form. Concerning the effect of Co and Ni as promoters, the higher activity for HDS and hydrogenation was archived using NiWS/SBA-15 catalysts [65].

Recently, Vradman *et al.* [65] studied the effect of deposition of the WS_2_ phase within the nanotubular channels of a pure silica SBA-15 substrate (at loadings up to 60 wt %) on the HDS activity of W/SBA-15 and Ni–W/SBA-15 sulfide catalysts. Sonication of a slurry containing SBA-15 material with a well-defined hexagonal crystalline structure in a W(CO)_6_-sulfur-diphenylmethane solution yielded an amorphous WS_2_ phase inside the mesopores. The Ni component was introduced into the WS_2_/SBA-15 precursor by impregnation with an aqueous solution of nickel acetate, drying under vacuum at room temperature and sulfidation. It was found that increasing the Ni content in the catalyst increased HDS activity up to a Ni/W ratio of about 0.4, followed by a slight decrease at a Ni/W ratio of 0.8. The optimized NiW/SBA-15 catalyst displayed 1.4-times higher HDS activity (DBT) compared with a sulfided commercial Co–Mo/Al_2_O_3_ catalyst [65].

Mural Dhar *et al.* [72] demonstrated that SBA-15-supported CoMo and NiMo catalysts are substantially more active in the HDS of thiophene than their counterparts supported on γ-Al_2_O_3_ prepared in a similar manner. The catalytic activity correlated well with the oxygen chemisorption results. Because only basic hydroxyl groups of SBA-15 are suitable for molybdenum anchoring, the oxygen chemisorption results suggested a patchy monolayer MoS_2_ formation on the surface of SBA-15 [72]. The number of these patches increases up to 8 wt % Mo, which contributes to the increase in anion vacancies at the edge of MoS_2_ and, in turn, to the catalyst activity. In addition, activity data of SiO_2_-supported catalysts revealed that the surface of SBA-15 differs from amorphous silica with respect to molybdenum interaction and hydrotreating functionalities [72].

Sampieri *et al.* [73] prepared MoS_2_/SBA-15 and MoS_2_/MCM-41 catalysts with Mo loadings as high as 20% without serious damage to the support structure. Whatever the Mo loading and the support, a high dispersion of the active phase was observed by TEM analysis. For SBA-15 supported catalysts, the MoS_2_ slabs appeared to be randomly oriented within its pores [73].

The performance for HDS of DBT and 4,6-DMDBT over NiMo/SBA-15 sulfide catalysts was reported by Ren *et al.* [74]. For these catalysts, the effect of varying the atomic ratio of Ni/Mo was studied. It was found that optimal Ni/Mo atomic ratio was 0.25. The catalyst with this optimized Ni/Mo atomic ratio exhibited excellent performance in the HDS reaction of the refractory 4,6-DMDBT. The HDS activity of this sample was superior to that of an industrial KF-848 catalyst tested under the same reaction conditions. The catalysts with a Ni/Mo atomic ratio in range 0.25–0.5 displayed comparable activity with this reference sample. These authors proposed that the enhancement of HDS activity of the SBA-15-based catalysts could be due to its higher hydrogenation ability with respect to that of a commercial catalyst [74].

An interesting attempt to improve HDS activity of NiMo/SBA-15 catalysts by the use of ternary Ni–Mo–W catalyst formulation has been reported by Mendoza-Nieto *et al.* [75]. The catalysts were prepared by co-impregnation (NiMoW) and by mechanical mixing (NiMo–NiW), and their activities were compared with those of bimetallic NiMo(W)/SBA-15 ones. It was found that the trimetallic NiMoW/SBA-15 catalyst prepared by co-impregnation showed the highest activity in the HDS of model compounds [75]. This is expected, because it is well established that Mo and W sulfided phases accelerate HDS reactions in a similar way, and the W-phase is less active than the Mo one [76].

In order to obtain more active HDS catalysts, the variation of the active phase was investigated by Gómez-Cazalilla *et al.* [35], which studied the HDS properties of novel chromium sulfide catalysts supported on Al-SBA-15 substrate. The novelty of this work was the use of chromium sulfide as the active phase, because the cost of chromium is much lower than that of Mo or W. Moreover, chromium is a relatively small atom, which can be loaded on supports in high proportions to achieve better catalytic activity without causing problems of dispersion. It was found that chromium sulfide-based catalysts supported on an Al-modified SBA-15 substrate exhibit good activity in the HDS of DBT at the high-pressure of hydrogen [35].

Jiménez-López and co-workers have found that ruthenium sulfide supported on pure SBA-15 and Zr-SBA-15 (Si/Zr molar ratio = 10) were more active in the HDS of dibenzothiophene than that supported on the Al-SBA-15 one (Si/Al molar ratio = 10) [49]. The dried catalysts were sulfided *in situ* with N_2_/H_2_S mixture (90%/10%) and without previous calcination. This is because the sulfidation in the absence of hydrogen allows generation of the pyrite phase along with the preparation of well dispersed and very active HDS catalyst [77]. The results reported highlight the important role that the material of support plays on the stability of the active phase, *i.e*., SBA-15 type mesoporous materials provided more stable ruthenium sulfide catalysts in the HDS of the DBT reaction than the MCM-41 mesoporous one, which was related to the higher diameter of pores leading to a better filing of them with ruthenium sulfide species.

In recent works, the same authors [78] investigated the effect of cesium addition to RuS_2_/SBA-15 catalysts in order to evaluate the role of Cs on the stabilization of the RuS_2 _phase. The influence of the cesium content, sulfiding temperature, as well as the cesium precursor was evaluated in the HDS of the DBT reaction at temperatures between 260 and 400 °C, upon high hydrogen pressure (3.0 MPa). The activity results revealed a negative effect with the addition of cesium, the most active catalyst being the one with the lowest content of Cs. In terms of selectivity, the presence of Cs induced the DBT transformation toward the direct desulfurization route, with selectivity to biphenyl close to 100%. From the catalyst activity-structure correlation, it was concluded that the addition of cesium to a RuS_2_/SBA-15 material does not favor a good dispersion of the RuS_2_ phase, and sulfur lability was inhibited in the presence of a large amount of Cs on the catalyst surface [78].

Lizama *et al.* [79] reported the effect of heteropoly-acids (H_3_PW_12_O_40_ and H_3_PMo_12_O_40_) as active phase precursors together with the effect of different promoters (Co against Ni) loaded on Mo(W)/SBA-15 catalysts on the performance HDS of 4,6-DMDBT. The catalyst characterization results demonstrated that the SBA-15-supported heteropoly-acids were well dispersed and maintained their characteristic Keggin structure once calcined at 350 °C for 2 h. Contrary to Co, the use of Ni as promoter led to a significant decrease in the temperature of the reduction of metal oxide species. Both Ni-promoted Mo/SBA-15 and W/SBA-15 catalysts showed high activity in the 4,6-DMDBT HDS reaction, which was substantially higher than that of the NiMo/Al_2_O_3_ reference sample. From these results, it was concluded that SBA-15 is a good substrate, and the heteropoly-acids as active phase precursors are promising for the preparation of novel Ni–Mo and Ni–W HDS catalysts [79].

A comparison study on the use of two different active phase precursors, Keggin-type heteropoly-acid, H_3_PW_12_O_40_ (HPW) and ammonium metatungstate in the synthesis of Ni-promoted W HDS catalysts, supported on SBA-15 or γ-alumina, was reported by the same authors [80,81]. It was found that the Keggin structure was preserved in the oxide precursor supported on SBA-15, whereas it was partially destroyed on γ-Al_2_O_3_, due to the strong interaction of parent HPW with this support. The catalysts supported on SBA-15 showed better catalytic performance than those supported on γ-alumina, and the use of heteropoly-acid precursor resulted in a further increase in catalytic activity. A comparative study of NiW catalysts was developed by loading a Ni salt of the 12-tungstophosphoric heteropoly-acid (H_3_PW_12_O_40_) on Al-, Ti- and W-modified SBA-15 [80,81]. From catalytic experiments, they concluded that Ti- and W-modified SBA-15 are more suitable for preparation of HDS catalysts than Al-SBA-15 supported catalysts when using Ni_3_/2PW_12_O_40_.

Dimitrov *et al.* [63] synthesized W-modified HMS and SBA-15 using sodium tungstate as the tungsten source. Both supports were impregnated with an aqueous solution of nickel salt of 12-tungstophosphoric acid Ni_3_/2PW_12_O_40_. The activity of NiW catalysts tested in the HDS of thiophene followed the order NiW/W-SBA-15 > NiW/W-HMS > NiW/γ-Al_2_O_3_. In the previous study [82], the NiW catalysts were prepared by loading a nickel salt of 12-tungstophosphoric heteropoly acid (H_3_PW_12_O_40_) on Al-, Ti- and W-modified SBA-15. The catalysts were tested in the HDS of thiophene at atmospheric pressure. It was found that Ti- and W-modified SBA-15 were more suitable for preparation of HDS catalysts than the Al-SBA-15 supported one [82].

#### 3.2.2. HDS Catalysts *ex* Thiosalts 

Thiosalt precursor decomposition is an interesting alternative method of catalyst preparation, which can provide an easy way to obtain a high level of sulfur content in the transition metal sulfide catalyst [83]. Moreover, contrary to the catalysts obtained by sulfidation of oxide precursors, the *in-situ* activation of thiomolybdate or thiotungstate precursors generally led to catalytic systems with a much higher specific surface area. In this respect, Huang *et al.* demonstrated that fully sulfided SBA-15-based Co-MoS_2_ [84,85] and Ni-MoS_2_ catalysts [86,87] could be easily obtained by decomposition of a thiomolybdate (ATM) complexes. The Co-MoS_2_ catalysts supported on SBA-15 showed a higher activity in the HDS of DBT reaction with a strong preference for the direct desulfurization pathway compared to a commercial CoMo/γ-Al_2_O_3_ catalyst [84]. An explanation proposed for the enhancement of the HDS activity involved the cooperative or synergistic effect of a CoMoS phase, the formation of Co_9_S_8_ species, as well as a large amount of coordinately unsaturated sites (CUS) formed during the thermal treatment of the ATM precursor.

A novel approach based on the use of already sulfided precursors (ammonium tetrathiomolybdate and cobalt diethyldithiocarbamate) was employed for studying the influence of the sequence of impregnation steps and the effect of different thiomolybdate precursors on the final catalytic response of binary Co–Mo/SBA-15 sulfide catalysts in the HDS of the DBT reaction [85]. It was found that the sequence of impregnation has no significant influence on the catalyst activity. On the contrary, the use of different thiomolybdate precursors significantly affects their HDS activity [85]. The CoMo/SBA-15 catalysts prepared from tetramethylammonium thiomolybdate (TMATM) exhibited lower activities in the HDS of the DBT reaction than their counterpart’s synthetized from ammonium thiomolybdate (ATM). The lower activity of the formers were explained as being due to the presence of pronounced pore blocking, as well as to the generation of big needle-like aggregates of the Co-MoS_2 _phase [85].

In good agreement with those observed for the Co–Mo/SBA-15 [85], it was found that the nature of the thiomolybdate complex shows a stronger influence on the MoS_2_ morphology than the impregnation procedure employed for the preparation of Ni-promoted MoS_2_/SBA-15 catalysts [86]. NiMoS_2_/SBA-15 catalyst derived from ammonium thiomolybdate (ATM) and a commercial NiMo/γ-Al_2_O_3_ catalyst exhibited similar activities in the HDS of DBT reaction [86]. High-resolution transmission electron microscopy investigations showed a high density of imperfect/disordered MoS_2_ nanocrystallites, which may contain a large number of coordinatively unsaturated sites being responsible for the relatively high HDS activity [87].

An additional study was performed by the same authors [87] to elucidate the influence of the catalyst activation method on the final catalyst behavior. It was found that the *in situ* activation performed in the presence of hydrocarbon solvent during the HDS of DBT was more beneficial for the preparation of NiMo catalysts with a high HDS performance compared to the *ex situ* activation mode using a N_2_/H_2_ (10% H_2_) gas flow at 500 °C. The authors concluded that low MoS_2 _stacking, small MoS_2_ slabs and a less pronounced pore blocking present in the *in situ* activated NiMo/SBA-15 catalysts, might be mainly responsible for the high HDS performance [87]. The *in situ* decomposition mode was claimed as a “softer” reducing atmosphere, which might represent a beneficial condition for the generation of the catalytic active “NiMoS” phase [87].

Alonso-Nuñez *et al.* [88] studied the influence of the activation atmosphere on the hydrodesulfurization of Co–Mo/SBA-15 catalysts prepared using already sulfided precursors (ammonium tetrathiomolybdate and cobalt diethyldithiocarbamate). Two sets of catalysts were synthesized using either a N_2_/H_2_ (10% H_2_) or a H_2_/H_2_S (15% H_2_S) atmosphere at three different temperatures of activation (450, 500 and 550 °C). The catalysts were tested in the HDS of DBT. Characterization by different techniques demonstrated that the use of already sulfided precursors led to a homogeneous dispersion of the active phase inside the SBA-15 channels. It was found that the N_2_/H_2 _activation procedure at 723 K allows obtaining optimized HDS active catalysts. A confinement effect of MoS_2_ slabs inside the SBA-15 channels led to a high selectivity along the direct desulfurization pathway [88].

### 3.3. Other SBA-15-Loaded Phases

#### 3.3.1. Metal Phosphides 

Transition metal phosphides display excellent activity for hydrodesulfurization and hydrodenitrogenation reactions [89,90,91,92,93,94,95,96,97,98,99,100], being the real alternative to sulfides in hydrotreating conditions. This is because the metal-rich transition metal phosphides (MP) or M_2_P (M = transition metal) compounds have metallic properties and larger intrinsic activities in HDS and hydrodesulfurization reactions with respect to classical sulfide-based catalysts [89]. They combine the properties of metals and ceramics, and thus, they are good conductors of heat and have high thermal and chemical stability [89]. Transition metal phosphides can be easy obtained by temperature-programmed reduction of corresponding oxides or chlorides [89,90]. However, during the HDS reaction, the formation of the phosphosulfide phases occurs, which are considered as the real active phases in HDS reactions catalyzed by phosphides [92]. Additionally, the catalyst reduction led to formation of a low amount of metal species, but apparently, this is not a drawback for the catalytic activity, as it was confirmed for alumina-supported molybdenum phosphide catalysts [91]. The reduction temperature affects the particle size of the active phase, which has significant influence on the HDS activity [5].

Contrary to the transition metal sulfides [27,28,29,30,31,32,33,34,35,36,71,72,73,74,75,76,77,78,79,80,81,82,83,84,85,86,87,88], studies on the use of SBA-15 substrate for supporting non-noble metal phosphides are not numerous [89,90,91,92,93,94,95,96,97,98,99,100]. In general, non-noble metal phosphides, such as Co, Ni, Mo or W, exhibited higher HDS activity than their sulfided counterparts [5]. In particular, the crystalline Ni_2_P is very active in HDS reaction, because of its higher intrinsic activity and dispersion, as compared to the other phosphides [94]. Moreover, during simultaneous HDS + HDN reactions, the HDS activity of Ni_2_P was not inhibited by N-containing compounds [94].

The P/Ni molar ratio was found to be the major parameter influencing the types of active phases formed [5,95,96]. In this sense, the study by Korányi *et al.* [95] demonstrated that HDS and HDN activities of the Ni_12_P_5_/SBA-15 and Ni_2_P/SBA-15 catalysts prepared with a low P/Ni initial ratio (P/Ni = 0.5) were always higher than those their counterparts prepared with a high P/Ni initial ratio (P/Ni = 2) [95]. The Ni_12_P_5_/SBA-15 and Ni_2_P/SBA-15 catalysts containing varying Ni loading (in the range 15–20 wt %) were prepared by co-impregnation, followed either by calcination at 500 °C or drying and, then, reduced up to 600 °C in hydrogen flow. The Ni_12_P_5_-containing SBA-15 catalysts exhibited higher hydrotreating activities than their counterparts containing the Ni_2_P active phase, only due to the higher dispersion of the former. The SBA-15-supported phosphide catalysts exhibited lower HDS and higher HDN activities than a commercial presulfided NiMo/Al_2_O_3_ reference catalyst [95].

The excellent performance in hydrotreating of coker light gas oil displayed SBA-15-supported nickel phosphide catalysts prepared by temperature-programed reduction of metal oxides [96]. It was found that the high surface area of SBA-15 support makes it a suitable carrier for better and uniform distribution of metal species. The Ni/P ratio played an important role in improving dispersion and, subsequently, reduction of the Ni and P species, which makes it active for hydrotreating reactions [96].

Wei *et al.* [97] prepared nickel phosphide/SBA-15/cordierite monolithic catalysts with different Ni contents (in the range 2.5–12.4 wt %) and an initial P/Ni molar ratio of 1:2. The nickel phosphide/SBA-15 was used for coating the cordierite support. The Ni_2_P phase and Ni_12_P_5_ phase were detected only when Ni content in the catalysts was high (9.9–12.4 wt %). The NiP/SBA-15/cordierite catalyst loaded with 9.9 wt % of Ni exhibited the high DBT conversion (99.2%) in the HDS of the DBT reaction at 380 °C. The DBT transformation over this sample proceeds mainly via the direct desulfurization reaction route. In the HDS of the DBT reaction at 380 °C, the activity of the monolithic catalyst was comparable with those of its nickel phosphide/SBA-15 powder, suggesting the absence of diffusion limitations in the monolithic catalyst [97].

The main drawback of the metal phosphides is their low active phase dispersion [5]. However, the dispersion of the Ni_2_P active phase on the surface of SBA-15 carrier could be enhanced by the addition of different promoters [98], as it was demonstrated for W-Ni_2_P/SBA-15 [99] and B-N_2_P/SBA-15 [100] catalysts.

#### 3.3.2. Metal Carbides 

Similarly to the metal phosphides, the properties of carbides are similar to those observed for the metals ones [5]. The carbides are formed by temperature-programmed reduction of the oxide precursors in CH_4_-H_2_ atmosphere, following by treatment with N_2_ containing a low quantity of oxygen (volume fraction lower than 1%) in order to passivate the carbides. A series of Mo_2_C/SBA-15 catalysts with different Mo contents (Si/Mo molar ratio = 30, 15, 7.5 and 1) were prepared by temperature-programmed carburization of their oxide precursors [101]. The catalyst characterization by different techniques demonstrated that the carburization process led to a change in the surface area and pore diameter. The MoO_3_ particles disintegrated during carburization of the oxide catalyst precursor, leading to formation of small Mo_2_C particles. Upon reaction conditions studied (temperature range from 175 to 400 °C; atmospheric pressure), the Mo_2_C/SBA-15 catalysts exhibited an excellent HDS activity. As expected, an increase in activity with an increase of Mo loading was observed [101]. Since it is known that carbides are easily transformed to sulfides in the presence of H_2_S [5], the high HDS activity of the Mo_2_C/SBA-15 catalysts is probably due to formation of molybdenum sulfide. Unfortunately, the HDS activity of Mo_2_C/SBA-15 catalysts was not referenced to any commercial HDS catalyst [101].

#### 3.3.3. Noble Metals

Supported noble metals are well known for their high activities for hydrogenation, hydrocracking and naphtha reforming. However, as compared with the transition metal sulfides [27,65,66,67,68,69,70,71,72,73,74,75,76,77,78,79,80,81,82,83,84,85,86,87,88], the effect of supporting noble metals on the SBA-15-based materials is much less studied [38,39,102]. This is because of their susceptibility to poisoning by sulfur, which arises from the fact that there is a strong adsorption of H_2_S on noble metal [103].

Since S-tolerance of noble metal catalysts could be enhanced by the use of acidic supports [103], the Al-SBA-15 substrate was employed for supporting noble metal (Pt, Pd, Rh and Ru) catalysts [38,39,102]. The catalyst activity was evaluated in the HDS of thiophene at 350 °C [38,39]. It was found that the Pt/Al-SBA-15 exhibited higher HDS activity than SBA-15-based Pd, Rh and Ru counterparts, and this activity was higher than that of commercial CoMo/Al_2_O_3_ HDS catalyst [38,39]. This was probably due to the dispersion of Pt on SBA-15 and remarkably enhanced by support Al grafting and because the acidity of Al-SBA-15 substrate was higher than that of pure SBA-15 [39]. The HDS activity of the Pt/Al-SBA-15 catalyst was remarkably enhanced with increasing Pt loading up to 5 wt % [39]. FTIR spectra of thiophene adsorbed on Al-SBA-15 indicate that thiophene molecules interact with Brønsted acid sites located on the surface of Al-SBA-15 substrate and that the strength of this interaction was stronger than that of SBA-15 [39].

Recently, Chandra Mouli *et al.* [102] evaluated the catalytic response of Pt/Al-SBA-15 systems in the hydrotreating of hydrotreated light gas oil (HLGO). The objective was to improve the cetane number of the LGO by ring opening of naphthenes. Commercial cracking Pt/HY catalyst was chosen to compare the ring opening activity with the studied Pt/Al-SBA-15 catalysts. Although the HLGO feed contained 1278 ppm of S, the HDS activity of those catalysts was not reported. It was found that the 1.5% Pt/Al-SBA-15 catalyst exhibited the largest improvement in the cetane index of HLGO, whereas reference (Pt/HY) catalyst was not able to improve the cetane index, mainly due to the formation of secondary cracking products. The activity results correlated well with the dispersion of the Pt on the Al-SBA-15 support surface [102].

## 4. Conclusions

On the basis of the present revision of the state-of-the-art on the use of SBA-15-based catalysts for hydrodesulfurization reactions, the following conclusions can be drawn:

(1) SBA-15 presents interesting textural properties that makes it attractive as support for hydrodesulfurization catalysts. Because of its lack of surface acidity and its low metal-support interaction, its modification with Al^3+^, Ti^4+^ or Zr^4+^ cations is needed. Moreover, it is emphasized at this point that SBA-15-based supports are investigated at the laboratory scale only because, for the refinery purpose, alumina substrate appears to be the cheapest solution. Thus, the challenge is to make the synthesis of SBA-15-based HDS catalysts more cost-effective.
(2) The incorporation of Al^3+^, Ti^4+^ or Zr^4+^ into SBA-15 improves the catalyst acidity, and active phase dispersion leads to enhancement of the HDS activity of SBA-15-supported catalysts with respect to the SBA-15- and Al_2_O_3_-supported ones. A similar effect was reported for SBA-15-based catalysts functionalized with mercaptopropyl groups [54].
(3) Preservation of the SBA-15 pore structure during support synthesis, modification and/or metal loading could be achieved by careful control of the pH. In order to maximize pore diameter without collapsing the pore structure of pure SBA-15, the pH should be close to 1. The catalysts with pores larger than 10 nm are needed for the hydroprocessing of heavy oils.
(4) The post-synthesis grafting of SBA-15 with Al^3+^, Ti^4+^ or Zr^4+^ ions is more effective than the direct synthesis method, because this leads to materials having a larger pore diameter and not to the limited diffusion of reactants and gives rise to the formation of Brønsted acid sites.
